# Optimizing Chronic Pain Treatment with Enhanced Neuroplastic Responsiveness: A Pilot Randomized Controlled Trial

**DOI:** 10.3390/nu13051556

**Published:** 2021-05-05

**Authors:** Steven Pratscher, Angela M. Mickle, John G. Marks, Harold Rocha, Felix Bartsch, Jeffrey Schmidt, Lazaro Tejera, Steven Garcia, Carlo Custodero, Federlin Jean, Cynthia Garvan, Alisa J. Johnson, Ralisa Pop, Anthony Greene, Adam J. Woods, Roland Staud, Roger B. Fillingim, Andreas Keil, Kimberly T. Sibille

**Affiliations:** 1 Pain Research and Intervention Center of Excellence, University of Florida, Gainesville, FL 32611, USA; Spratscher@ufl.edu (S.P.); john.marks@urology.ufl.edu (J.G.M.); alisa.johnson@ufl.edu (A.J.J.); rpop@dental.ufl.edu (R.P.); rfillingim@dental.ufl.edu (R.B.F.); 2 Department of Community of Dentistry, University of Florida, Gainesville, FL 32611, USA; amickle@dental.ufl.edu; 3 Department of Psychology, University of Florida, Gainesville, FL 32611, USA; hrocha1@usf.edu (H.R.); felixbartsch@ufl.edu (F.B.); stevenegarcia1012@gmail.com (S.G.); akeil@ufl.edu (A.K.); 4 College of Health Professions and Sciences, University of Central Florida, Orlando, FL 32816, USA; jeffreyschmidt2012@gmail.com; 5 Department of Interdisciplinary Medicine, University of Bari, 70125 Bari, Italy; lazarotejera96@gmail.com (L.T.); c.custodero@ufl.edu (C.C.); 6 Department of Aging & Geriatric Research, University of Florida, Gainesville, FL 32611, USA; federlinjean@gmail.com; 7 Department of Anesthesiology, University of Florida, Gainesville, FL 32611, USA; cgarvan@anest.ufl.edu; 8 Counseling and Wellness Center, University of Florida, Gainesville, FL 32611, USA; agee@ufl.edu; 9 Department of Clinical and Health Psychology, Center for Cognitive Aging and Memory Clinical Translational Research, University of Florida, Gainesville, FL 32611, USA; woodsaj@gmail.com; 10 Department of Medicine, University of Florida, Gainesville, FL 32611, USA; roland.staud@medicine.ufl.edu

**Keywords:** chronic pain, neuroplasticity, intermittent fasting, glucose, osteoarthritis, non-invasive interventions

## Abstract

Chronic pain affects mental and physical health and alters brain structure and function. Interventions that reduce chronic pain are also associated with changes in the brain. A number of non-invasive strategies can promote improved learning and memory and increase neuroplasticity in older adults. Intermittent fasting and glucose administration represent two such strategies with the potential to optimize the neurobiological environment to increase responsiveness to recognized pain treatments. The purpose of the pilot study was to test the feasibility and acceptability of intermittent fasting and glucose administration paired with a recognized pain treatment activity, relaxation and guided imagery. A total of 32 adults (44% W, 56% M), 50 to 85 years of age, with chronic knee pain for three months or greater participated in the study. Four sessions were completed over an approximate two-week period. Findings indicate the ability to recruit, randomize, and retain participants in the protocol. The procedures and measures were reasonable and completed without incident. Participant adherence was high and exit interview feedback positive. In summary, the pilot study was feasible and acceptable, providing the evidence necessary to move forward with a larger clinical trial.

## 1. Introduction

Chronic pain is a highly prevalent and disabling condition associated with decreased quality of life [1,2,3] and increased morbidity and mortality [4,5]. Although chronic pain occurs throughout the lifespan, its incidence increases with age [6]. Knee osteoarthritis (OA), a degenerative joint disease, is characterized by chronic pain, inflammation, and loss of function and is among the leading causes of disability in older adults [7,8,9]. The prevalence and corresponding public health impact of knee OA are expected to increase in the coming years [7,10,11].

Chronic pain not only diminishes quality of life and health but also alters brain structure and function [12,13,14,15,16,17,18]. For example, chronic pain conditions, such as knee OA, are associated with changes in central sensitization, gray matter volume, and brain network dynamics [12,15,16,19,20,21,22,23]. These observed differences in the brain and nervous system are thought to represent neuroplastic changes that may underpin or maintain a chronic pain state [13,20,24,25,26].

Some clinical interventions reverse brain functional and structural changes associated with chronic pain [24,27,28,29,30]. Additionally, a strong body of evidence indicates the brain is plastic across the lifespan and non-invasive strategies (e.g., exercise, intermittent fasting, intermittent hypoxia, brief hyperthermia, or hypothermia exposure) can enhance neuroplastic responsiveness [31,32,33,34,35]. Thus, pairing strategies that promote neuroplasticity with existing chronic pain treatments might optimize their clinical effectiveness [35,36]. Two non-invasive, inexpensive, and easy to implement neuroplastic strategies that may bolster chronic pain treatments are intermittent fasting and glucose administration [36]. 

Intermittent fasting has been shown to promote neuroplasticity and may have long-term brain and health benefits [37,38,39]. Intermittent fasting is a type of time-restricted eating with minimal or no caloric intake for periods of time as few as 12 hours [40,41,42]. A 16 to 18 hours fasting regimen is among the most popular types of intermittent fasting for humans and has been shown to be acceptable, adherable, and beneficial for older adults [37,40,41,43,44,45]. Although some dietary patterns have been investigated for their impact on reducing chronic pain [46,47], intermittent fasting may serve as a standalone treatment and/or adjuvant treatment for people with or at risk of developing chronic pain. 

Glucose administration is another dietary strategy indicated to improve cognitive performance, learning, memory, and neuroplasticity, including in older adults [48,49,50]. Although administering glucose to a chronic pain population with high rates of obesity may seem counterintuitive, studies of glucose administration typically use a small amount of glucose (30–50 g), equivalent to about a glass of orange juice, tethered to the timing of an intervention [36,50]. Circulating blood glucose levels peak around 30 min post-administration and tend to return to baseline after about 2 hours [50], which provides a window of opportunity to capitalize on the learning and memory facilitation effects of glucose by implementing a pain treatment during that time. 

Electroencephalogram (EEG) measures provide the ability to evaluate neurophysiological changes and quantify cortical plasticity within and across different experimental conditions. As intermittent fasting and glucose administration are indicated as promoting neuroplasticity, EEG measures could be informative. Specifically, somatosensory evoked potentials, as used in the current study, have been effective in assessing experience-induced plastic changes in the somatosensory cortex [51]. In regard to selecting a recognized pain treatment strategy for the current study, several criteria were important. The strategy needed to be brief, standardized, repeatable, generalizable across chronic pain conditions, and applicable and adaptable to the EEG component of the study. Relaxation and guided imagery are well-recognized pain treatment strategies that are frequently included in pain coping skills training programs [52,53,54,55]. Thus, we developed a relaxation and guided imagery script to serve as the standardized exposure activity [56]. 

The purpose of the present study was to evaluate the feasibility and acceptability of the pilot randomized controlled trial “Optimizing Chronic Pain Treatment with Enhanced Neuroplastic Responsiveness” (OPTIMIZE). Specifically, intermittent fasting and glucose administration interventions were paired with a relaxation and guided imagery activity in individuals with chronic knee pain with or at risk for knee osteoarthritis. A pilot study provides the opportunity to identify any modifications necessary to successfully conduct a larger, subsequent RCT [57]. We hypothesized good feasibility in terms of participant recruitment and retention as well as intervention adherence and acceptability.

## 2. Materials and Methods 

### 2.1. Study Design and Setting

The pilot randomized trial was designed to test the feasibility and acceptability of intermittent fasting and glucose administration, compared to a normal eating control group, for older adults with chronic knee pain. Participants completed 4 study sessions over approximately 14 days. Due to the nature of the study, it was not possible to blind the participants to the intervention. Only the EEG personnel were blinded to the study randomization. 

### 2.2. Ethics

The University of Florida Institutional Review Board approved the study, and a Data Safety Monitoring Board reviewed the study bi-annually. Prior to enrollment, all participants provided verbal and written informed consent. This study follows CONSORT reporting guidelines [58] and was pre-registered on ClinicalTrials.gov (NCT02681081).

### 2.3. Participants

Participants were adults 50–85 years old in the community of Gainesville, Florida, with chronic knee pain lasting for at least three months prior to enrollment. Participants were excluded from the study if they had a concurrent medical condition that could confound outcome measures or limit their ability to participate completely in the protocol, including neurological conditions (e.g., Parkinson’s disease, multiple sclerosis, and/or seizures); history of a head injury or stroke; diabetes or taking medications to control blood sugar; mental health issues resulting in hospitalization or outpatient treatment in the past year, and/or psychotropic medication use; current or history of treatment for alcohol or other substance abuse; cognitive function ≤ 22 on the Mini-Mental Status Exam (MMSE); pregnancy; high baseline fasting blood sugar (plasma glucose ≥ 7 mmol/L); persisting blood pressure > 150/95 or a heart condition such as a prior heart attack, heart surgery (including a stent), frequent chest pain or heart failure; or, inability to complete the EEG portion of the study.

### 2.4. Procedures

#### 2.4.1. Recruitment and Screening

Potential participants were recruited through flyers (e.g., in community, clinics, and research settings), referrals, and local study registries. Interested participants were pre-screened over the phone and provided information about the study. Participants who were successfully pre-screened were invited for the first study visit to provide informed consent and undergo additional screening. Overall recruitment goals were to (1) recruit 20 participants per group (60 total) in a two-year period; (2) have a retention rate of 70% with at least 14 participants completing all 4 study sessions in each group (42 total).

To assess fasting blood sugar levels at the first session, potential participants were asked to refrain from eating and drinking caloric beverages after 8 pm the evening prior and exercising in the previous 24 hours. Session 1 study screening procedures involved a health history review with a current list of medications, a short test of thinking and memory (MMSE), a blood draw for blood glucose levels, heart rate and blood pressure measurements, and a pregnancy test, if applicable. With inclusion criteria confirmed, study eligible participants completed the baseline assessments, which included primary study measures as described below and noted in Table 1.

#### 2.4.2. Randomization

Randomization was determined using a statistically generated block formula. Following the completion of the screening session, participants were randomized to one of three groups: intermittent fasting, glucose administration, or normal eating control. Participants were informed about the group to which they were randomly assigned and provided instructions regarding the next three study sessions. 

#### 2.4.3. Interventions

The intermittent fasting group was instructed to fast for 16 hours prior to sessions 2–4 [44,59,60]. Specifically, participants were asked not to consume food or beverages other than non-caloric beverages or black coffee after 6 or 7 pm the evening prior to each session. On non-session days, participants were instructed to follow their normal dietary intake.

The glucose administration group was instructed to fast for two hours prior to sessions 2–4. At the beginning of sessions 2–4, blood glucose level was assessed, then participants consumed 30 g of a pharmaceutical grade liquid glucose administered by a clinical research nurse [50,61,62]. Blood glucose levels were re-assessed approximately 30 min after glucose administration. On the non-session days participants were instructed to follow their normal dietary intake.

The normal eating control group was instructed to follow their normal dietary intake throughout the course of the study. 

#### 2.4.4. Sessions 2–4

Following session 1, participants were scheduled for three additional sessions within a 10-day period. Upon arrival, participants were asked about their current level of knee pain, any changes in health and medication since the last visit, level of distress (subjective units of distress scale, SUDS), and the last time food or drink was consumed. Blood pressure and heart rate were collected at the beginning and end of each session. Blood glucose levels were also assessed at the start and end of each session with a blood draw during visits 1, 2, and 4 and a finger prick test during visit 3. During visit 4, participants repeated the measures collected at baseline. They also completed an exit survey regarding their participation in the study. To encourage attendance, participants were contacted before each study visit to remind them of their appointment. 

#### 2.4.5. Standardized Exposure Activity

Relaxation and guided imagery are well-established treatments for coping with chronic pain [52,53,54,56]. A recorded and standardized script served as the repeated exposure activity in the study. All participants, regardless of intervention group, participated in a 15 min audio-recorded relaxation and guided imagery activity at each session. The activity was comprised of four components: relaxation breathing, guided imagery [56,63], positive postural statements (e.g., smile), and the presentation of validated positive emotion words [64].

### 2.5. Measures

Measures described are limited to those addressing feasibility and acceptability.

#### 2.5.1. Demographic and Health Information

Participants baseline demographics included sex, ethnicity/race, highest level of education, household income, satisfaction with living standards and income, marital status, number living in the household, work status, and insurance coverage. Participants completed a baseline health screen including blood pressure, mean arterial pressure and heart rate, and anthropometrics including head, waist, and hip circumference, height, and weight. Current and past comorbidities were assessed from a pre-specified list including high blood pressure, heart disease, cancer, diabetes, asthma/breathing problems, kidney disease, thyroid problem, stroke, seizure, chronic pain, neurological disorder, depression, other mental health conditions or health problems. Other health behaviors, including smoking, weekly exercise frequency, and current medication usage were also collected.

#### 2.5.2. Clinical Pain Measures

Western Ontario and McMaster Universities Osteoarthritis Index (WOMAC) [65]. The WOMAC measures lower extremity pain and function in persons with OA over the past 48 h. The WOMAC has 24 items rated on a 5-point Likert scale (0 = None to 4 = Extreme) that measure pain (0–20 score), stiffness (0–8), and physical function (0–68). Sub-scales are summed for an overall total score (0–96). Higher scores represent worse symptoms and physical disability. The WOMAC was collected at sessions 1 and 4.

Graded Chronic Pain Scale (GCPS) [66]. The GCPS assesses the severity of knee pain in the past three months. The GCPS is scored on two scales: characteristic pain intensity score (0–100) and pain-related disability score (0–100). Higher scores indicate greater characteristic pain intensity and pain-related disability. The GCPS was collected at session 1.

Chronic Pain Stage-Knee. The chronic pain stage index was used for pain phenotyping and included questions regarding pain frequency, intensity, time, and total pain sites (FITT). Frequency of knee pain was assessed as either persistent (1 point) or intermittent (0 points). Intensity of pain was measured using the GCPS characteristic pain intensity score. Frequency of knee pain was measured in months. Additionally, participants were asked to mark the areas where they have experienced pain on more days than not for the past 3 months from a pre-specified list of 14 bilateral body sites (hands, arms, shoulders, neck, head/face/jaw, chest, stomach, pelvis, upper back, lower back, knees, legs, feet/ankles, and other). Median splits were calculated for intensity, time, and total pain sites with a point given for those above the median [67,68]. The total chronic pain stage-knee score was the sum of the FITT dimensions and ranged from 0 to 4 where 0 = low pain and 4 = high/severe pain. This FITT measure has been shown to associate with biomarkers of immune and metabolic functioning, cellular aging, and brain structure [67,68,69].

#### 2.5.3. Experimental Pain Measures

Order of experimental pain testing (i.e., punctate and pressure) and site (e.g., knee, hand, other) was randomized across participants. Experimental pain measures were collected at sessions 1 and 4.

Punctate mechanical testing. Punctate mechanical stimuli were delivered to the most painful knee and the back of the ipsilateral hand using a 300 g nylon monofilament. This test involved delivering two trials of a single stimulus and a series of 10 repeated stimuli, then and asking participants to report if they experienced pain and the intensity of pain on a scale from 0 to 10.

Pressure pain threshold. Pressure pain threshold was assessed on the most painful knee and two sites ipsilateral to the tested knee—the lateral epicondyle and the trapezius muscle. A constant rate of pressure (1 kg/s) was applied using a handheld algometer. The participant was instructed to indicate when the sensation first became painful.

#### 2.5.4. Affect, Mood, and Stress Measures

Positive and Negative Affect Schedule (PANAS) [70]. The PANAS is a 20-item scale that assesses positive (e.g., excited, inspired) and negative (e.g., nervous, irritable) affect. At session 1, participants rated “in general” responses to 10 positive words and 10 negative words on a 5-point Likert scale from 1 (very slightly or not at all) to 5 (extremely). During sessions 2–4, participants were asked to rate the same words about how they were feeling “right now”. Scores are divided into a positive affect and a negative affect scale from 10 to 50 with higher scores indicating higher levels of affect.

Perceived Stress Scale (PSS) [71]. The PSS is a 10-question survey that measures thoughts and feelings during the last month on a scale from 0 (never) to 4 (very often). Scores range from 0 to 40 with higher scores representing greater perceived stress. The PSS was completed at sessions 1 and 4.

Subjective Units of Distress (SUDS). The SUDS was used at the beginning and end of each study session to assess levels of distress on a 0–10 scale ranging from 0 (no distress) to 10 (highest distress ever felt) [72,73].

#### 2.5.5. Cognitive Measures

Hopkins Verbal Learning Test (HVLT) [74]. The HVLT is a brief cognitive screener assessing verbal learning and memory. The HVLT consists of a recall and recognition portion. There are 4 sub-scores: total recall, delayed recall, retention, and recognition discrimination index, which are then transformed to a *t*-score.

Trail Making Test Parts A and B (Trails A and B) [75,76,77]. Trails A and B is a pen and paper cognitive screening tool. Participants are provided a copy of Trails A, which consists of 25 circles distributed over a sheet of paper which are numbered 1–25. Participants are asked to draw a line connecting the numbers in ascending order. In Part B, the circle includes both number (1–13) and letters (A–L). Participants are then asked to draw a line connecting the numbers and letters, alternating between the two in numerical and alphabetical order. Both tasks are timed with >78 s for Trails A and >273 s for Trails B indicative of impaired performance.

#### 2.5.6. Biological Measures

Blood Glucose Level. Blood glucose levels were measured with the YSI 2300 STAT Plus Glucose and l-Lactate Analyzer (YSI Incorporated, Yellow Springs, OH, U.S.A.). Fasting blood glucose was collected with a blood draw at the first session. Blood glucose levels were also measured twice during sessions 2 and 4 and with a finger prick using a standard glucometer for session 3. 

#### 2.5.7. EEG Measures

Somatosensory evoked potentials and spectral power in the EEG alpha band (8–13 Hz) were collected using a 32-electrode array system (ActiChamp, BrainProducts, Gilching, Germany). Data were collected during dedicated periods of sessions 1 and 4, as follows: A 3 min EEG resting block during an initial resting phase (90 s eyes closed, 90 s eyes open, in this order) was followed by a somatosensory stimulation block, a relaxation and guided imagery activity, another tactile stimulation block, and a final resting block as previously described [78].

Somatosensory evoked steady state potentials (SSSPs). These signals were collected during the somatosensory stimulation block. To this end, a mechanical haptic stimulator (TSD190; BIOPAC, Galeta, CA, USA) with an internal electromagnetically actuated plunger (1.5 mm diameter) was programmed to stimulate the skin at a temporal rate of 2.77 Hz at a non-painful level. The stimulator was attached to each knee and wrist, in sessions 1 and 4, during the stimulation blocks indicated above for a duration of 180 s at each location. EEG signals were filtered, artifact-corrected, and then projected into the frequency domain using discrete Fourier transform according to standard methods (see Rocha et al., 2020). The signal-to-noise of the spectral power peak at the stimulation frequency of 2.77 Hz served as the dependent variable.

Alpha power: Alpha power was extracted from EEG signals collected during the initial resting phase (eyes closed, eyes open) and during the relaxation and guided imagery procedure. To this end, the EEG signal was first segmented into 2 s epochs, and epochs with artifacts were rejected. Then, discrete Fourier transform (DFT) was conducted for each artifact-free segment, and the resulting DFT spectra were averaged across segments to yield a mean frequency spectrum for each experimental period of interest. Paralleling SSSP analysis, alpha power was then extracted as the ratio of alpha power (spectral power between 8 and 13 Hz) relative to the rest of the spectrum, averaged across all posterior sensor locations.

#### 2.5.8. Additional Explanatory Variables

Pittsburgh Sleep Quality Index (PSQI) [79]. The PSQI measures sleep quality and disturbance over the past month. Total scores range from 0 to 21, with higher scores indicating worse sleep quality.

Center for Epidemiologic Studies Depression Scale (CES-D) [80]. The CES-D measures depressive symptoms over the past week. Scores range from 0 to 60, with higher scores indicating more depressive symptomatology.

Patient-Reported Outcomes Measurement Information Symptom Measures System (PROMIS) Anxiety 7a [81]. The PROMIS anxiety measures emotional distress in the past 7 days on with 8 items rated on a 5-point Likert scale from 1 (never) to 5 (always). Scores range from 7 to 35, with higher scores indicating greater anxiety.

Patient-Reported Outcomes Measurement Information Symptom Measures System (PROMIS) Depression SF8b [81]. The PROMIS depression scale consists of 8 items on a 5-point Likert scale ranging from 1 (never) to 5 (always). Higher scores are consistent with increased depression.

Exit Questionnaire. Exit questions were developed to characterize acceptability of the two dietary interventions and study-related experiences. Questions were included specific to each intervention group (“e.g., I would complete a 16 hour fast at least two days a week if it improved my overall health” or “I would consume glucose and complete a two hour fast once a week if it improved my overall health”) and were rated on a 5-point Likert scale (1 = Strongly disagree, 5 = Strongly agree). Participants were also given the opportunity to provide comments about their involvement in the study. This feedback provided information about potential adaptations for a subsequent study.

### 2.6. Statistical Analyses

Descriptive statistics, including means and standard deviations (SD) for continuous variables and frequencies and percentages for categorical variables, were used to describe participant demographics, baseline characteristics, and feasibility and acceptability outcomes. Intervention groups were tested for differences in baseline characteristics using Mann–Whitney U test for continuous data and Fisher’s exact test for categorical data. Non-parametric statistics were chosen given the small sample size. Results are organized by sub-headers of recognized key components to include in the evaluation of pilot studies [57,82,83,84]. Feasibility was evaluated specific to recruitment, randomization, retention, and adherence. Acceptability was measured by safety (adverse events), pre/post session self-reported distress, and the exit questionnaire.

## 3. Results

### 3.1. Recruitment

Participants were primarily recruited from posted advertisements in the community, other studies, and by word of mouth. A total of 135 community-dwelling adults were screened for eligibility. Of those, 34 were ineligible, 27 were not interested, and 33 did not respond or were unable to participate because of their schedule. Of the 43 individuals who completed the in-person screening assessment, 11 were excluded after further screening due to medication or health concerns. Thus, 32 participants were randomly assigned to the intermittent fasting group (*n* = 11), glucose administration group (*n* = 11), or normal eating control group (*n* = 10). Screening began in March of 2016 and concluded in December 2017. Recruitment was paused for a number of months during the study timeframe due to delays in access to EEG equipment as a result of a relocation of the lab to a new facility on campus. The study was concluded when the sample size to assess feasibility and acceptability for a pilot study was obtained.

### 3.2. Randomization

Participant demographics are presented in Table 2 for the 32 participants who were randomized. The age range of participants was 51–80 years (mean = 63.9 years, SD = 8.37 years) with 62.5% of the participants being 60–80 years of age. The participants were predominantly white (75%), non-Hispanic (94%), males (56%), and had at least some college education (72%).

### 3.3. Retention

**Figure 1:**
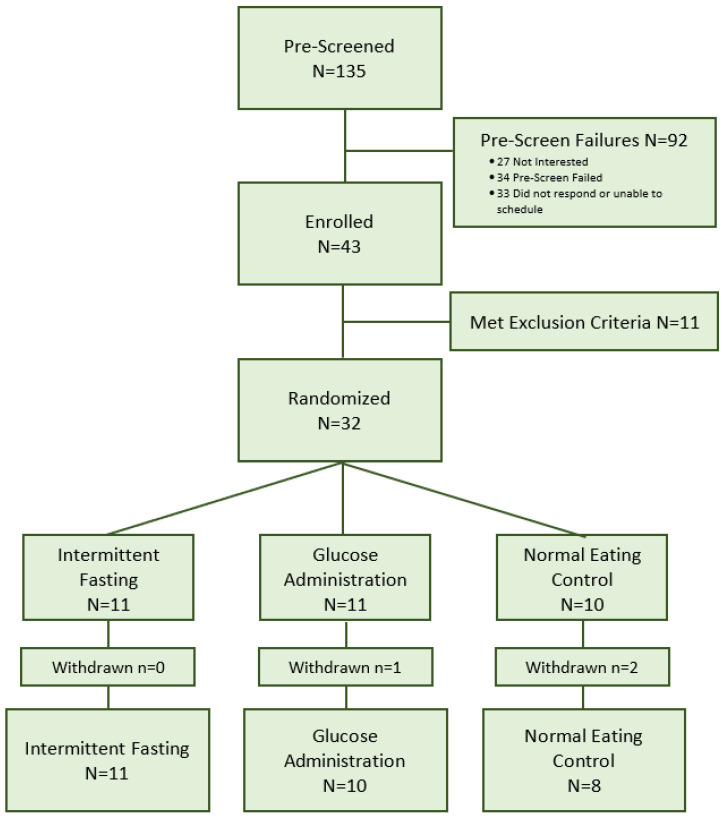
CONSORT Flow Chart.

The retention rate was high (90.6%) with 3 out of 32 participants being withdrawn from the study before the final session (*n* = 2 for unrelated health issues, *n* = 1 for travel distance). Figure 1 depicts the recruitment and enrollment process.

### 3.4. Adherence

#### 3.4.1. Adherence to Intervention

All 29 participants who completed the study attended all four sessions. At the start of each session, participants self-reported the last time they ate and drank. Participants reported adhering to the fasting time length specific to their assigned group (i.e., 16 h for the intermittent fasting group and 2 h for the glucose administration group). Blood glucose levels were also assessed at the start and end of each session as an objective measure of adherence as well as indicator of intervention effect (glucose administration). As expected, blood glucose levels were lower in the intermittent fasting and glucose administration groups compared to the normal eating control and consistent with the clinical range for a fasting level, Table 3 [85]. As the normal eating control group participants were able to adhere to their normal eating patterns, some participants reported their last mealtime as the evening prior to the session, resulting in blood glucose levels similar to the other groups in session 2 and 4.

#### 3.4.2. Adherence to Protocol

Participants completed all assessment procedures. Missing data were limited to those measures collected after session 1 from participants withdrawn from the study. Table 4 displays baseline characteristics on primary measures by intervention group and indicates missing data.

### 3.5. Acceptability

No adverse events were reported. Procedures were completed without incident. Physical and emotional responses were assessed. Specifically, in addition to collecting physiological measures each session, subjective units of distress (SUDS) scores were assessed at the beginning (T1) and conclusion (T2) of each study session (see Table 5). Participants in all groups reported a decrease in average levels of distress at the conclusion of each session, with most scores indicating no distress at all.

In the exit interview, participants answered questions regarding the acceptability of the intervention. Most participants in the intermittent fasting group (Table 6, Panel A) and glucose administration group (Table 6, Panel B) reported the intervention was not difficult to implement and that it would be a sustainable intervention if it could improve their health.

## 4. Discussion

The intention of the current pilot study was to examine the feasibility and acceptability of pairing intermittent fasting and glucose administration with a recognized pain treatment intervention, relaxation and guided imagery in individuals with chronic knee pain with or at risk for OA. As hypothesized, the results indicated good feasibility and acceptability of the study design and procedures. The findings are encouraging and provide support for carrying out a larger, fully powered randomized controlled trial (RCT). A review of key feasibility and acceptability components and potential utility of intermittent fasting and glucose administration are discussed in further detail below.

### 4.1. Feasibility

Recruiting from the community was a successful strategy to obtain a sample of older community-dwelling adults with chronic knee pain with or at risk for knee OA. Most participants were recruited from community advertisements without the involvement of recruitment services. The length of time to meet the minimum sample size was delayed due to a pause in recruitment resulting from the relocation and delays in accessing the EEG lab. Nevertheless, there was strong positive interest in the study. The block randomization approach resulted in minimal differences in baseline characteristics between the intervention groups even with the small sample size. A significant difference was indicated in WOMAC measures between intervention groups; however, the WOMAC is a self-reported measure of knee pain, stiffness, and physical limitation in the prior 48 h. The GCPS captures knee pain and function over the prior three months and no group differences were observed. Thus, when randomizing for a chronic pain condition, limitations in short-term pain indices need to be considered and may not serve as a strong measure to evaluate randomization. Further, the 90% participant retention rate was encouraging given participation involved attending 4 in-person sessions over a 10- to 14-day period. Phone call reminders were effective in promoting session attendance.

### 4.2. Adherence

In regard to adherence to the interventions, blood glucose levels at the start and conclusion of each session aligned with anticipated ranges of individuals fasting for 16 and 2 h and with variable eating patterns. Additionally, participants self-reported adherence to their respective intervention group. A few individuals in the normal eating control group regularly did not eat breakfast, which lowered the overall mean blood glucose levels in that group. For future studies, individuals in the control group should be asked to eat breakfast to reduce potential overlap with the fasting group. The implementation of the protocol occurred without incident. All measures were collected as planned and missing data were minimal. As all assessments occurred in-person, there were no issues collecting measures across all four sessions.

A number of findings support the acceptability of the interventions. In addition to the observed adherence and compliance patterns, the majority of participants from both the intermittent fasting and glucose administration groups reported that following the intervention was not difficult and that they would continue if it would improve their health. Furthermore, comments from the exit interview indicated that a large proportion of participants reported enjoying being involved in the study and expressed interest in participating in future studies.

### 4.3. Limitations, Implications, and Future Directions

This pilot study allowed for the opportunity to evaluate participant response to the protocol, interventions, procedures, and measures. Limitations and possible opportunities for improvements for future studies follow. First, session 1 required approximately four hours for completion. Sessions beyond four hours would likely exceed a comfortable time range for participants and the research team. One option to reduce the length of the first session would be to have participants complete some of the self-report measures in advance, which could then be reviewed during the session. Another option would be to complete the screening and baseline measures in week one and begin the intervention component of the study the following week. Second, due to changes in accessibility to the EEG equipment, we experienced periods of time where we were not able to recruit and run study participants. Designing the study to include access to compatible backup equipment could prevent study completion delays and disruptions. Third, the study was not dosed for clinical benefit (limited to 3 intervention sessions in a 10-day period). Since this was a proof-of-concept study and was not intended to demonstrate clinical efficacy, we cannot be certain the levels of attrition would be the same in a future trial dosed for clinical benefit with a longer follow-up period. Finally, although not noted as a concern by participants directly, the term “fasting” is often interpreted as an extended period without food and may discourage participation by some individuals. Evaluating participant’s perceptions of the term “fasting” and exploring alternative terms may be beneficial.

Our findings indicated that we were able to recruit older adults with chronic pain to participate in a short-term intervention. These participants reported a willingness to engage in a longer intermittent fasting and glucose administration intervention. We also demonstrated that pain sensory testing and EEG methods were feasible to implement and will serve to measure possible neuronal mechanisms associated with these interventions to create better target therapeutics. Additionally, we were able to show a standardized relaxation and guided imagery protocol was well tolerated and would serve as a useful exposure activity in future investigations. Although the current study was not dosed to reduce pain, there is strong rationale to investigate intermittent fasting as a possible preventative strategy, standalone treatment, and/or as a combined therapy with a pain treatment. Specifically, intermittent fasting may be beneficial or reduce risk factors and comorbidities associated with chronic pain based on research showing that intermittent fasting increases weight loss [86,87], slows aging and age-related diseases [40,41], enhances stress resistance and resilience [43,88], improves learning and cognitive function [45], and decreases both peripheral inflammation and neuroinflammation [37,41].

Future investigations are also warranted for glucose administration. There is a strong body of evidence supporting the benefits of glucose administration in promoting learning, memory, and neuroplasticity. Although glucose administration differs from intermittent fasting with respect to potential direct health benefits, it may serve a beneficial role in populations where intermittent fasting would not be recommended or would be difficult to implement (children) or in settings where a combined treatment strategy might optimize the gains of an intervention (physical therapy for a cognitively impaired population). Thus, there are a number of important avenues for future research specific to the role of intermittent fasting and glucose administration in the optimization of treatments for chronic pain conditions.

First-line treatments for knee OA include education and self-management therapies with pharmacological management and surgery to follow [7]. Given the costs and risks of pharmacological management and surgery, there is a need for non-invasive, cost-effective treatments for persistent knee OA pain [89]. Intermittent fasting and glucose administration are two strategies which may contribute toward improving chronic pain treatments. Our findings show the pilot randomized controlled trial of intermittent fasting and glucose administration was feasible and acceptable, providing the evidence necessary to move forward with a larger clinical trial.

## Figures and Tables

**Table 1:** Timetable of primary study measures.

	Session 1	Session 2	Session 3	Session 4
**Screening and Baseline Measures**				
Demographics	X			
MMSE	X			
Health History	X			
**Cardiovascular Measures**				
Heart Rate	X	X	X	X
Mean Arterial Pressure	X	X	X	X
Blood Pressure	X	X	X	X
**Anthropometric Measures**				
Head Circumference (EEG)	X			
Height/Weight	X			
Waist/Hip Circumference	X			
**Clinical Pain Measures**				
WOMAC	X			X
GCPS	X			
Chronic Pain Stage-Knee	X			
**Experimental Pain Measures**				
Punctate Stimuli	X			X
Pressure Stimuli	X			X
**Affect, Mood, Stress Measures**				
PANAS	X(Trait)	X(State)	X(State)	X(State)
PSS	X			X
SUDS	X	X	X	X
**Cognitive Measures**				
HVLT	X			X
Trails A and B	X			X
**Biological Measures**				
Glucose Blood Draw	X	X		X
Glucose Finger Stick			X	
**EEG Measures**				
Alpha power	X			X
Alpha Block	X			X
SST	X			X
**Additional Explanatory Variables**				
PSQI		X		
CESD	X			X
PROMIS Anxiety and Depression	X			X

**Table 2:** Participant demographics.

Variable	Intermittent Fasting (*n* = 11)	Glucose Administration (*n* = 11)	Normal Eating Control (*n* = 10)	Total (*n* = 32)
Age, years, mean (SD)	65.15 (6.73)	62.52 (9.04)	63.96 (9.81)	63.88 (8.37)
Sex, female *n* (%)	4 (36%)	4 (36%)	6 (60%)	14 (44%)
Ethnicity, Hispanic *n* (%)	1 (9%)	1 (9%)	0 (0%)	2 (6%)
Race, *n* (%)				
White	8 (73%)	7 (64%)	9 (90%)	24 (75%)
Non-White	3 (27%)	4 (45%)	1 (10%)	8 (25%)
Education, *n* (%)				
High school degree	4 (36%)	2 (18%)	3 (30%)	9 (28%)
Two-year college degree	3 (27%)	3 (27%)	1 (10%)	7 (22%)
Four-year college degree	4 (36%)	5 (45%)	5 (50%)	14 (44%)
Doctoral degree	0 (0%)	1 (9%)	1 (10%)	2 (6%)
Marital status, *n* (%)				
Married	4 (36%)	3 (27%)	5 (50%)	12 (38%)
Widowed	0 (0%)	1 (9%)	0 (0%)	1 (3%)
Divorced	2 (18%)	3 (27%)	3 (30%)	8 (25%)
Never married	5 (45%)	2 (18%)	2 (20%)	9 (28%)
Living with partner	0 (0%)	2 (18%)	0 (0%)	2 (6%)
Body mass index (BMI), kg/m^2^, mean (SD)	30.58 (5.61)	32.37 (11.78)	33.40 (11.93)	32.08 (9.88)
Baseline glucose level, mean (SD)	86.91 (6.85)	88.36 (8.35)	88.80 (4.32)	88.00 (6.61)

**Table 3:** Blood glucose levels across sessions by intervention group.

	Session 2		Session 3		Session 4	
Session Mean (SD)	T1	T2	T1	T2	T1	T2
Intermittent Fasting	87.5 (4.9)	85.6 (7.1)	93.1 (11.2)	95.6 (11.6)	87.7 (7.6)	86.2 (7.3)
Glucose Administration	86.2 (9.7)	132.7 (23.1)	95.1 (11.3)	161.5 (25.2)	87.2 (12.1)	146.3 (22.8)
Normal Eating Control	87.7 (14.4)	90.1 (8.0)	112.2 (24.7)	107.3 (9.5)	91.0 (10.2)	86.5 (11.8)

Glucose levels measured in mg/dL by blood draws in session 2 and 4 and by finger stick in session 3. Note: T1 was collected upon arrival to the session and T2 was collected approximately 30 min after glucose administration for participants in the glucose administration group.

**Table 4:** Primary baseline measures by intervention group.

Variable	Intermittent Fasting (*n* = 11) Mean (SD)	Glucose Administration (*n* = 11) Mean (SD)	Normal Eating Control (*n* = 10) Mean (SD)	Total (*n* = 32)	*p*-Value
**Clinical Pain**					
WOMAC Pain	4.91 (2.21)	8.09 (2.12)	6.60 (4.06)	6.53 (3.10)	0.046
WOMAC Stiffness	2.55 (1.37)	3.64 (1.21)	3.30 (2.06)	3.16 (1.59)	0.28
WOMAC Physical Function	17.27 (7.76)	27.73 (9.63)	21.50 (14.66)	22.19 (11.46)	0.049
WOMAC Total	24.73 (9.98)	39.45 (12.31)	31.40 (19.60)	31.87 (15.20)	0.047
GCPS Intensity	50.30 (16.50)	55.45 (15.93)	45.67 (24.09)	50.63 (18.83)	0.65
GCPS Disability	40.61 (24.53)	43.64 (25.19)	48.67 (34.51)	44.17 (37.49)	0.87
Chronic Pain Stage	1.64 (1.36)	2.45 (1.13)	1.70 (0.95)	1.94 (1.19)	0.16
**Experimental Pain**					
Pressure Pain Threshold (kg)					
Forearm	2.99 (1.28)	3.03 (1.05)	2.70 (0.76)	2.91 (1.04)	0.86
Lateral Knee	4.42 (1.18)	4.58 (1.20)	4.29 (1.32)	4.44 (1.20)	0.76
Medial Knee	4.09 (1.45)	4.58 (1.20)	4.16 (1.45)	4.10 (1.39)	0.99
Trapezius	4.76 (1.51)	4.59 (1.19)	3.54 (0.71)	4.32 (1.28)	0.08
Mechanical/Punctate Pain Rating (0–100 scale)					
Hand Single	7.23 (8.98)	9.27 (5.41)	6.85 (6.03)	7.81 (6.87)	0.35
Hand Series	12.82 (13.01)	24.90 (18.43)	12.6 (11.73)	16.9 (15.45)	0.13
Knee Single	11.41 (13.08)	24.32 (19.75)	17.35 (17.51)	17.7 (17.31)	0.17
Knee Series	21.86 (15.67)	42.18 (26.36)	32.85 (20.99)	32.28 (22.47)	0.10
**Affect and Stress**					
Positive Affect	38.04 (5.20)	37.82 (5.74)	37.10 (5.04)	37.69 (5.20)	0.96
Negative Affect	12.46 (3.42)	13.00 (2.49)	13.40 (4.70)	13.25 (3.42)	0.92
PSS	12.55 (3.93)	11.82 (4.73)	11.50 (6.10)	11.97 (4.82)	0.78
**Cognitive**					
HVLT Delayed	47.55 (12.01)	46.73 (7.67)	52.78 (7.10)	48.77 (9.42)	0.35
HVLT Discrimination	48.73 (9.52)	44.36 (10.86)	54.11 (8.34)	48.74 (10.18)	0.07
HVLT Recall	49.55 (13.03)	45.73 (9.33)	49.67 (16.10)	48.23 (12.58)	0.55
HVLT Retention	46.91 (13.16)	51.45 (9.03)	52.11 (11.61)	50.03 (11.25)	0.73
Trials A	32.8 (11.07)	30.82 (8.28)	27.8 (6.70)	30.56 (8.89)	0.40
Trails B	91.18 (31.03)	76.82 (26.99)	63.0 (26.95)	77.44 (29.85)	0.09
**EEG**					
Resting Alpha-Eyes Closed (SNR)	1.65 (0.92) ^a^	2.26 (1.12) ^a^	1.68 (0.71) ^a^	1.87 (0.97) ^a^	0.13
Resting Alpha-Eyes Open (SNR)	1.31 (0.58) ^a^	1.62 (0.93) ^a^	1.25 (0.36) ^a^	1.40 (0.68) ^a^	0.34
Open–Closed Alpha Blocking (difference SNR)	−0.34 (0.95) ^a^	−0.63 (2.26) ^a^	−0.44 (0.86) ^a^	−0.47 (0.88) ^a^	0.60
Somatosensory potentials (SNR; knees)	2.23 (0.85)	2.27 (0.87)	2.36 (0.93)	2.32 (0.86)	0.97
**Additional Explanatory Variables**					
PSQI	10.73 (4.27)	12.00 (2.87) ^a^	11.89 (3.37) ^a^	11.50 (3.51)	0.70
CES-D	7.36 (4.27)	6.27 (8.46)	7.20 (5.87)	6.94 (6.26)	0.37
PROMIS Depression	12.00 (3.41)	11.09 (3.83)	10.50 (3.95)	11.22 (3.66)	0.37
PROMIS Anxiety	13.53 (2.95)	13.64 (4.59)	13.20 (2.82)	13.46 (3.46)	0.99

^a^ = Missing data from one participant.

**Table 5:** Subjective units of distress across sessions by intervention group.

	Session 2		Session 3		Session 4	
Mean (SD)	T1	T2	T1	T2	T1	T2
Intermittent Fasting	1.45 (0.82)	0.73 (0.65)	1.27 (0.79)	0.73 (0.79)	2.36 (1.50)	1.45 (1.44)
Glucose Administration	1.30 (0.82)	0.80 (0.92)	1.50 (0.71)	0.30 (0.48)	1.10 (0.32)	0.70 (0.48)
Normal Eating Control	1.44 (0.73)	0.44 (0.73)	1.67 (0.71)	0.38 (0.52)	1.25 (0.46)	1.13 (0.64)

Note: T1 was collected upon arrival to each session and T2 was collected at the conclusion of each session.

**Table 6:** Exit interview responses for intermittent fasting and glucose administration interventions.

**Intermittent Fasting**		
	**Mean (SD)** **1 = Strongly Disagree,** **3 = Neither, 5 = Strongly Agree**	**Median** **(Minimum–Maximum)**
Completing the 16 h fast was difficult for me	2.45 (1.29)	3.00 (1–4)
I would complete a 16 h fast at least two days a week if it improved my overall health	4.27 (1.27)	5.00 (1–5)
I noticed I had less pain when I fasted	2.64 (0.51)	3.00 (2–3)
I noticed that I was less irritable when I fasted	2.82 (0.87)	3.00 (1–4)
Fasting made tasks at my work, school or at home more difficult	2.27 (1.01)	2.00 (1–4)
I had more energy than normal on fasting days	2.64 (0.92)	3.00 (1–4)
I had difficulty falling asleep, staying asleep, or waking up on nights following a fasting day	2.18 (1.08)	2.00 (1–5)
**Glucose Administration**		
	**Mean (SD)** **1 = Strongly Disagree,** **3 = Neither, 5 = Strongly Agree**	**Median** **(Minimum–Maximum)**
Fasting for two hours and consuming glucose was difficult for me	1.80 (1.03)	1.50 (1–4)
I would consume glucose and complete a two hour fast once a week if it improved my overall health	4.60 (0.52)	5.00 (4–5)
I noticed I had less pain while participating in this study	2.50 (0.85)	2.50 (1–4)
I noticed that I was less irritable over the last week	3.30 (0.82)	3.00 (2–5)
Participating in this study improved my ability to function better at work, school or at home	3.30 (0.95)	3.00 (2–5)
I had more energy than normal over the past week	2.90 (0.74)	3.00 (2–4)
I had difficulty falling asleep, staying asleep, or waking up on nights over the past week	2.50 (1.27)	2.00 (1–5)
